# Prognostic value and gene regulatory network of CMSS1 in hepatocellular carcinoma

**DOI:** 10.3233/CBM-230209

**Published:** 2024-04-15

**Authors:** Cheng Chen, Caiming Wang, Wei Liu, Jiacheng Chen, Liang Chen, Xiangxiang Luo, Jincai Wu

**Affiliations:** a Department of Hepatobiliary and Pancreatic Surgery, Hainan General Hospital, Hainan Affiliated Hospital of Hainan Medical University, Haikou, Hainan, China; b Department of Operation Room, Hainan General Hospital, Hainan Affiliated Hospital of Hainan Medical University, Haikou, Hainan, China; c Department of Breast, Guangzhou Red Cross Hospital, Guangzhou Red Cross Hospital of Jinan University, Guangzhou, Guangdong, China

**Keywords:** CMSS1, hepatocellular carcinoma, . bioinformatics, diagnosis, prognosis

## Abstract

**BACKGROUND:**

Cms1 ribosomal small subunit homolog (CMSS1) is an RNA-binding protein that may play an important role in tumorigenesis and development.

**OBJECTIVE:**

RNA-seq data from the GEPIA database and the UALCAN database were used to analyze the expression of CMSS1 in liver hepatocellular carcinoma (LIHC) and its relationship with the clinicopathological features of the patients.

**METHODS:**

LinkedOmics was used to identify genes associated with CMSS1 expression and to identify miRNAs and transcription factors significantly associated with CMSS1 by GSEA.

**RESULTS:**

The expression level of CMSS1 in hepatocellular carcinoma tissues was significantly higher than that in normal tissues. In addition, the expression level of CMSS1 in advanced tumors was significantly higher than that in early tumors. The expression level of CMSS1 was higher in TP53-mutated tumors than in non-TP53-mutated tumors. CMSS1 expression levels were strongly correlated with disease-free survival (DFS) and overall survival (OS) in patients with LIHC, and high CMSS1 expression predicted poorer OS (P< 0.01) and DFS (P< 0.01). Meanwhile, our results suggested that CMSS1 is associated with the composition of the immune microenvironment of LIHC.

**CONCLUSIONS:**

The present study suggests that CMSS1 is a potential molecular marker for the diagnosis and prognostic of LIHC.

## 1. Introduction

Liver hepatocellular carcinoma (LIHC) is the predominant pathological type of primary liver cancer, accounting for approximately 90% of all liver cancers [1]. Currently, the main treatment options for LIHC are surgical resection, liver transplantation, interventional therapy, ablative therapy, targeted therapy and immunotherapy [2]. Due to the lack of early symptoms and effective diagnostic markers, most patients with LIHC are usually advanced at the time of initial diagnosis and lose the opportunity for radical resection. Therefore, there is an urgent need to identify more new and reliable biomarkers to aid in early diagnosis and early treatment.

RNA binding proteins (RBPs) are powerful and extensive regulators in cells, which participate in gene expression regulation at multiple levels by specifically recognizing RNA, such as RNA stability, pre-mRNA splicing, translation, etc. [3]. RBPs have important regulatory roles in cell development, metabolism and various diseases [4, 5]. More and more attention has been paid to the involvement of RNA-binding proteins in the regulation of tumor-related biological processes, including the regulation of apoptosis, epithelial-to-mesenchymal transition (EMT), and autophagy [6, 7, 8, 9, 10]. CMSS1 (Cms1 Ribosomal Small Subunit Homolog) is a protein-coding gene that encodes an RNA-binding protein. In recent years, more and more attention has been paid to the regulatory role of RNA-binding proteins in malignant tumors [11]. Therefore, we investigated the expression landscape of CMSS1 in LIHC, the functional enrichment analysis of CMSS1, the impact of CMSS1 expression levels on patient prognosis, the relationship between CMSS1 and the immune microenvironment of LIHC, and so on. Thus, the results of this study may provide new molecular markers for the diagnosis and prognosis of LIHC.

## 2. Materials and methods

### 2.1 Expression of CMSS1 in LIHC and its effect on prognosis

This study was conducted by investigating the expression of CMSS1 in LIHC cancer and normal tissues through the Gene Expression Profile Interaction Analysis (GEPIA) database (http://gepia.cancer-pku.cn/index.htm) [12]. GEPIA has data of 369 cancer tissue samples from LIHC patients and 160 normal samples. The “single gene” module of GEPIA was used to analyze the mRNA expression of CMSS1 in cancer tissues of LIHC patients and normal tissues. Multi-gene comparative analysis was then performed using the “Multi-gene comparison” module of GEPIA and the “LIHC” dataset. Kaplan-Meier curves were used to show disease-free survival (DFS) and overall survival (OS). Patients with LIHC were divided into high and low expression groups based on median transcripts from RNA-seq (TPM), and data were displayed by hazard ratio (HR) and 95% confidence.

### 2.2 UALCAN analysis

UALCAN (http://ualcan.path.uab.edu) has RNA sequencing data of 31 different cancers and can be used to analyze the relationship between gene expression and clinicopathological characteristics [13]. In this study, we analyzed the clinicopathological characteristics of CMSS1 and LIHC, including the relationship between tumor stage, pathological grade, gender and TP53 mutation status through the UALCAN platform, and the analysis process is as follows. During the analysis, we selected “CMSS1” as the gene to be studied and “LIHC” as the cancer type.

### 2.3 LinkedOmics analysis

LinkedOmics database (http://www.linkedomics.org /login.php) is an online database of 32 cancers [14]. In this analysis, we selected the RNAseq data of CMSS1 in the “TCGA_LIHC” cohort, and Pearson correlation test was used to analyze the expression correlation between CMSS1 and other genes. In this study, genes that were significantly associated with CMSS1 expression in the LIHC cohort were analyzed using the LinkFinder module. The results are shown in the volcano and heat maps. GO [cell composition (CC), biological process (BP) and molecular function (MF) analysis], KEGG signaling pathway analysis, miRNA target enrichment and transcription factor target enrichment were performed for differentially associated genes using genomic enrichment analysis (GSEA).

### 2.4 Relationship between CMSS1 and immune infiltration microenvironment in LIHC

TIMER is an easy-to-use database for analyzing the immune infiltration of various tumors [15]. We used the TIMER algorithm to estimate the relationship of CMSS1 with the immune microenvironment in LIHC. The gene module was used to assess the relationship of CMSS1 with the infiltration of six immune cells (CD4+ T cells, CD8+ T cells, macrophages, B cells, dendritic cells and neutrophils).

**Figure 1.**
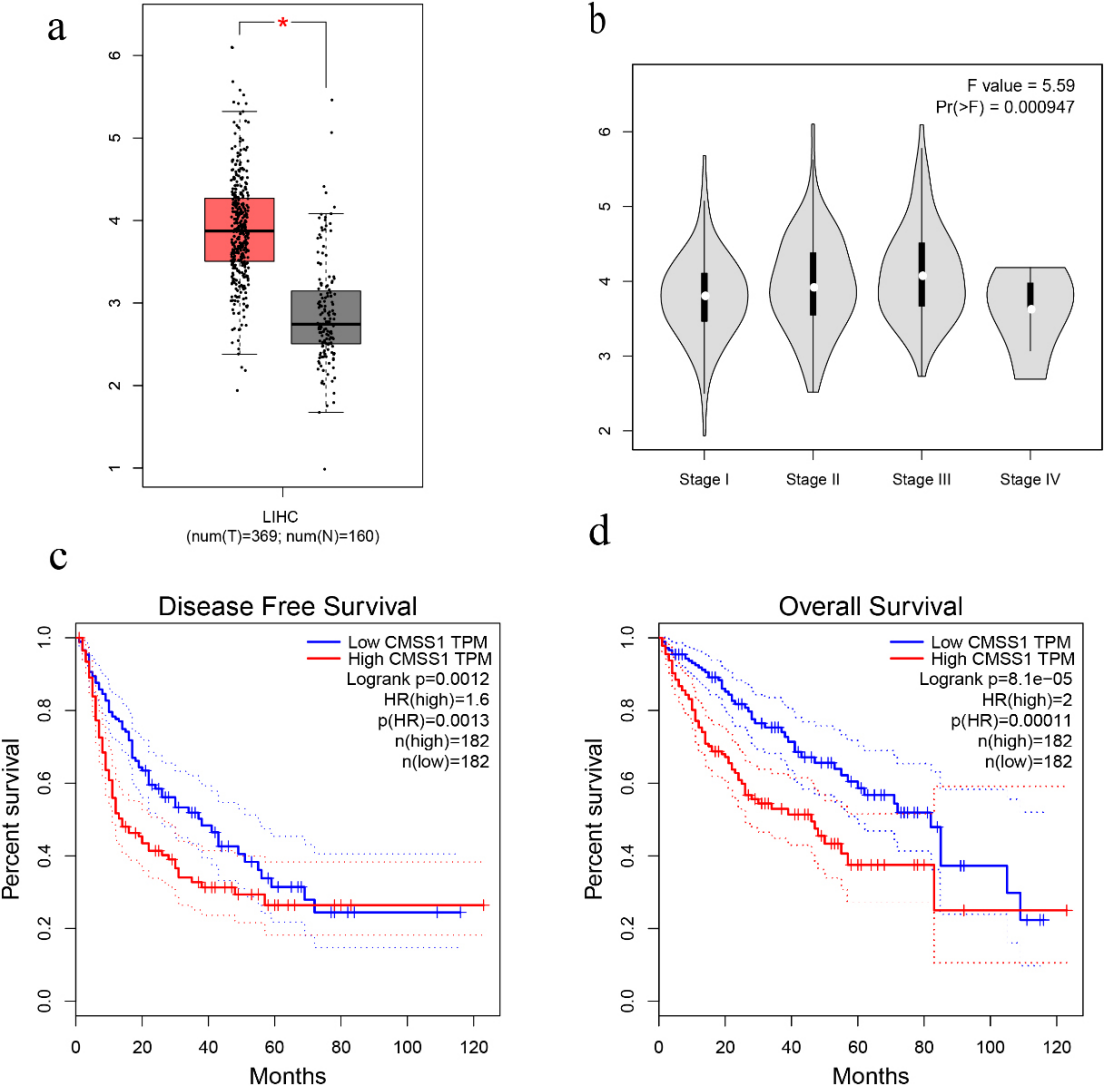
The expression level of CMSS1 in LIHC and its influence on prognosis. (a) Box plot showing CMSS1 mRNA levels in LIHC and normal tissue. (b) Violin diagram showed the expression of CMSS1 in different stages of LIHC. (c) Disease-free survival curve based on CMSS1 high and low expression grouping. (d) Overall Survival Curve Based on CMSS1 High and Low Expression Grouping.

### 2.5 ProteinAtlas analysis

ProteinAtlas (https://www.proteinatlas.org/) is an open access resource for human proteins that allows querying the expression of proteins in various tissues and cells, including immunohistochemical profiles of protein expression in different tumor tissues and normal tissues. Therefore, we analyzed CMSS1 expression at the protein level in clinical tumor samples and normal liver tissues in ProteinAtlas. After typing “CMSS1” in the search box, we can see an overview of the gene and its detailed expression in ten sections, such as Tissue, Brain, Pathology, etc. If we click on one of the sections, such as Tissue, we can see its expression in various tissues of the human body, including normal liver tissue. Then, we clicked into one of the “PATHOLOGY” sections to see its expression in various cancer tissues of human body. Then, we clicked on “Liver Cancer” to see the IHC staining with different antibodies.

**Figure 2.**
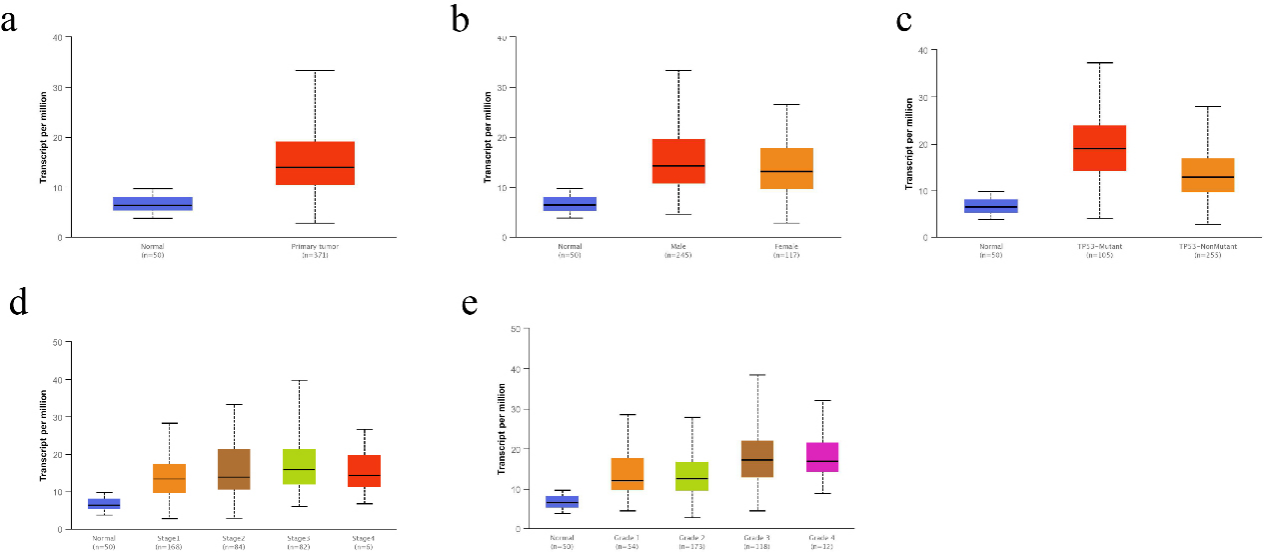
Correlation of CMSS1 transcript levels with clinicopathological features in patients with LIHC. (a) The Box-plot showed the relative expression of CMSS1 in normal and LIHC tissues. (b) The Box-plot showed the expression of CMSS1 in LIHC patients of different genders. (c) The Box-plot showed the relative expression of CMSS1 in normal or LIHC tissues with different TP53 mutation status. (d) The Box-plot showed the expression of CMSS1 in different stages of LIHC. (e) The Box-plot showed the relative expression of CMSS1 in LIHC tissues of different histological grades. Data are mean ± SE. *, P< 0.05; **, P< 0.01; ***, P< 0.001.

### 2.6 Patient samples and qRT-PCR

Twenty paired frozen hepatocellular carcinoma tissues and normal tissue samples were stored in a refrigerator at negative eighty degrees. The experimental protocol was approved by the Ethics Committee of Hainan General Hospital (NO.2022-274). We extracted total RNA from hepatocellular carcinoma tissues and normal tissues using the Universal RNA Extraction Kit (TaKaRa, Kyoto, Japan), and examined the quantity and quality of isolated RNA using a Nanodrop (Thermo Fisher Scientific). A cDNA library was constructed using Prime-Script RT Master Mix Kit (TaKaRa). The cDNA library was constructed using the Prime-Script RT Master Mix Kit (TaKaRa). qRT-PCR was performed using the SYBR Green Master Mix Kit (TaKaRa). β-actin was used as a control.

### 2.7 Statistical analysis

Differences between LIHC cancer tissues and normal tissues were analyzed by t-test, and correlations were analyzed by Spearman’s correlation coefficient. LIHC patients were divided into CMSS1-high and CMSS1-low groups using median TPM as the threshold. Kaplan-Meier curves were used to show the differences in DFS and OS. The log-rank test was used to compare the differences between groups. P< 0.05 was defined as a significant difference.

## 3. Results

### 3.1 Expression of CMSS1 in LIHC

We investigated the correlation between the expression level of CMSS1 and the clinicopathological characteristics of LIHC patients. Comparative analysis of 369 liver cancer tissue and normal tissue samples from the GEPIA database revealed that the level of CMSS1 mRNA was significantly higher in LIHC than in normal tissues (Fig. 1a). The UALCAN database further validated that the expression level of CMSS1 was significantly higher in LIHC tissues than in normal liver tissues (Fig. 2a). These results strongly suggest that CMSS1 can be used as a diagnostic biomarker for hepatocellular carcinoma. Subsequently, we also found that CMSS1 was closely associated with aggressive clinicopathological features. Interestingly we observed that CMSS1 was expressed significantly higher levels in male LIHC patients than in female patients (Fib. 2b). We found progressively higher and statistically different expression levels of CMSS1 from stage I to stage III (P< 0.001), where the sample size of stage IV was too small to show this trend (Fig. 1b and Fig. 2d). We also found that CMSS1 expression was also significantly higher in LIHC tissues with high histological grade than in patients with low histological grade (Fig. 2e).

Interestingly, we also found that CMSS1 expression was significantly higher in TP53-mutated tumors than in non-TP53-mutated LIHC (Fig. 2c). Finally, survival analysis further confirmed that CMSS1 expression was significantly associated with recurrence-free survival (DFS, Fig. 1c) and overall survival (OS, Fig. 1d) of patients. This suggests that CMSS1 can be used not only as a biomarker for the diagnosis of LIHC, but also as a biomarker for predicting the prognosis of LIHC.

**Figure 3.**
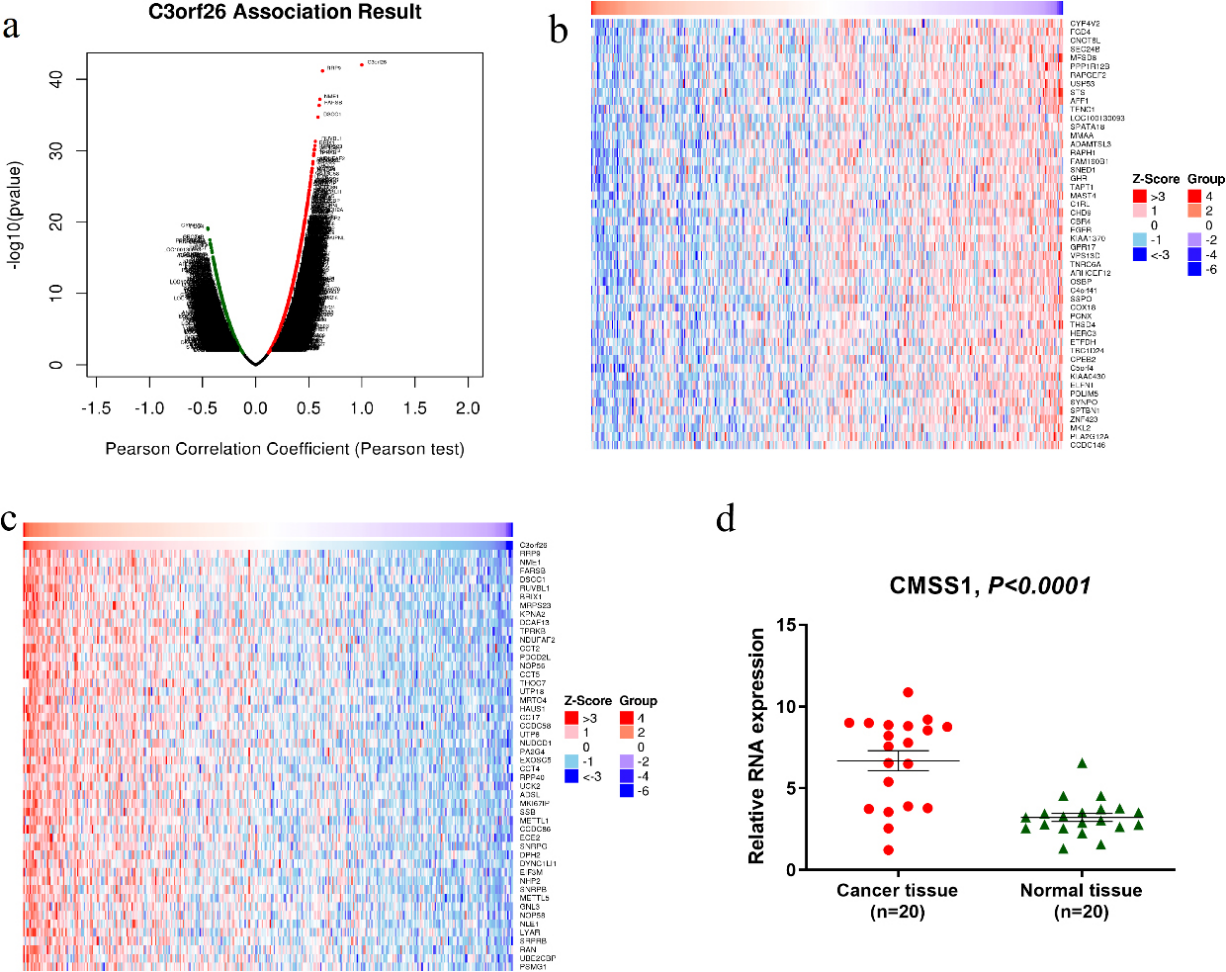
Differentially expressed genes related to CMSS1 in LIHC (C3orf26 is the alias of CMSS1 gene). (a) Pearson test to analyze the genes associated with CMSS1 expression in LIHC (b, c) Heat maps shows the genes positively and negatively associated with CMSS1 in LIHC (top 50). Red indicates positively correlated genes and green indicates negatively correlated genes. (d)The mRNA expression of CMSS1 in HCC tissues was significantly higher than that in normal liver tissues.

### 3.2 GO and KEGG pathway analysis of genes related to CMSS1 expression in LIHC

As shown in the volcano plot (Fig. 3a), CMSS1 was positively or negatively correlated with the expression of a large number of genes in LIHC with statistical differences. The heat map showed that the top 50 genes were positively and negatively correlated with CMSS1, respectively (Fig.3b and c). Among them, RRP9, NME1, FARSB, DSCC1 and RUVBL1 were the top 5 most significantly positively associated genes (Fig. 3c), while CYP4V2, FGD4, CNOT6L, SEC24B and MFSD8 were the top 5 most significantly negatively associated genes (Fig. 3b). Then we examined the expression of CMSS1 mRNA in 20 pairs of hepatocellular carcinoma tissues and normal liver tissues adjacent to the carcinoma, and the results showed that the expression level of CMSS1 in the carcinoma tissues was significantly higher than that in the normal tissues (Fig. 3d). These results suggest that CMSS1 may have an extensive gene regulatory network.

**Table 1:** CMSS1-related miRNAs and transcription factor-target networks in liver cancer (LinkedOmics)

Enriched category	Gene set	Leading-Edge-Num	FDR
miRNA Target	TGCACTT, MIR-519C, MIR-519B, MIR-519A	155	0
	CTTTGCA, MIR-527	103	0
	GGCACTT, MIR-519E	51	0
	TAATGTG, MIR-323	62	0
	GTGCAAT, MIR-25, MIR-32, MIR-92, MIR-363, MIR-367	123	0
Transcription Factor Target	V$FREAC4_01	59	0
	V$E2F1DP1_01	69	0.00017309
	V$E2F1DP2_01	69	0.00017309
	V$E2F4DP2_01	69	0.00017309
	SGCGSSAAA_V$E2F1DP2_01	60	0.00023079

**Figure 4.**
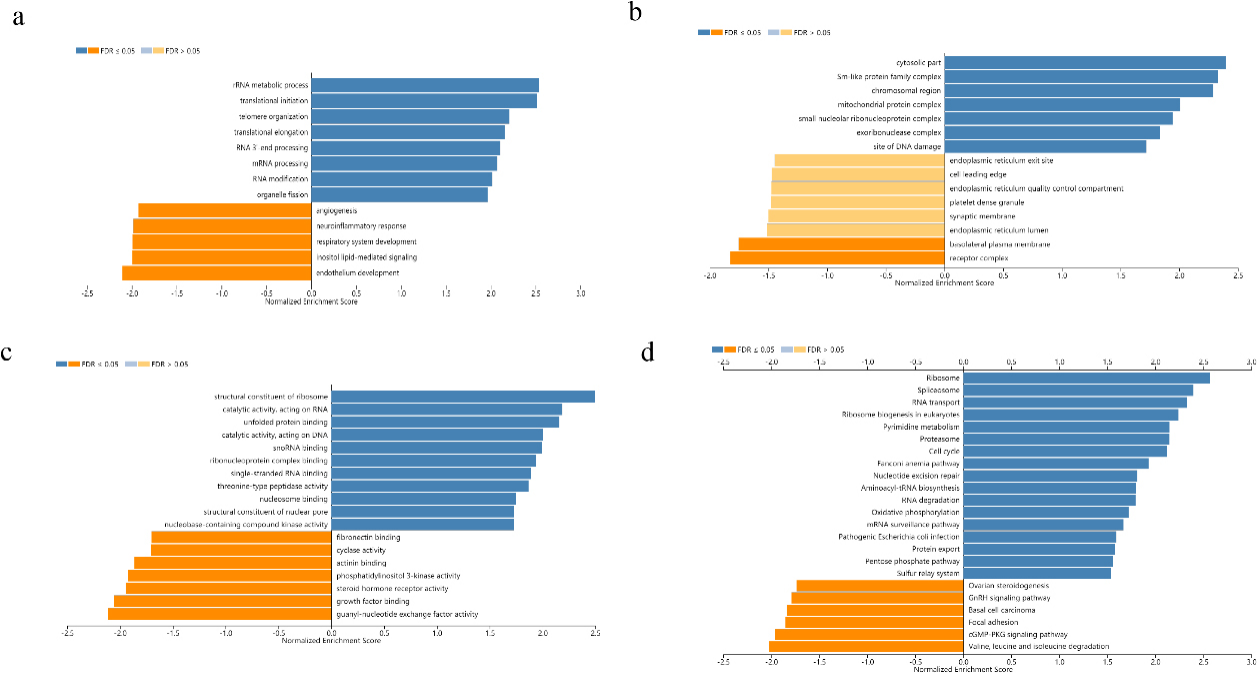
GO annotation and KEGG pathway analysis of CMSS1 expression related genes in LIHC. (a) Biological processes; (b) Cellular components; (c) Molecular function; (d) KEGG pathway analysis.

Biological process analysis suggested that the biological functions of differentially expressed genes positively correlated with CMSS1 were concentrated in the biogenesis of ribonucleoprotein complexes, rRNA metabolism, ncRNA processing, translation initiation, RNA localization and RNA catabolism (Fig. 4a). Analysis of cellular components reveals that differentially expressed genes associated with CMSS1 play structural roles in the ribosome, cytoplasmic fraction, pre-ribosome and spliceosome complexes (Fig. 4b). Molecular function analysis showed that the biological functions of differentially expressed genes that were positively correlated with CMSS1 were concentrated in the structural components of ribosomes, RNA binding, catalytic activity acting on RNA, and unfolded protein binding. (Fig. 4c). KEGG analysis revealed that differentially expressed genes positively correlated with CMSS1 were significantly enriched in the ribosome, spliceosome, RNA transport, ribosome biogenesis and DNA replication pathways in eukaryotes (Fig. 4d).

**Table 2:** Multifactorial analysis of the effect of CMSS1 and immune infiltrating cells on the survival of LIHC

	Coef	HR	95%CI_l	95%CI_u	P value	Sig
B_cell	-7.282	0.001	0.000	0.515	0.031	*
CD8_Tcell	-5.328	0.005	0.000	0.797	0.041	*
CD4_Tcell	-3.468	0.031	0.000	14.262	0.267	
Macrophage	4.023	55.862	0.425	7336.081	0.106	
Neutrophil	1.063	2.896	0.000	148763.156	0.848	
Dendritic	4.322	75.352	2.407	2358.977	0.014	*
CMSS1	0.571	1.771	1.331	2.356	0.000	***

**Figure 5.**
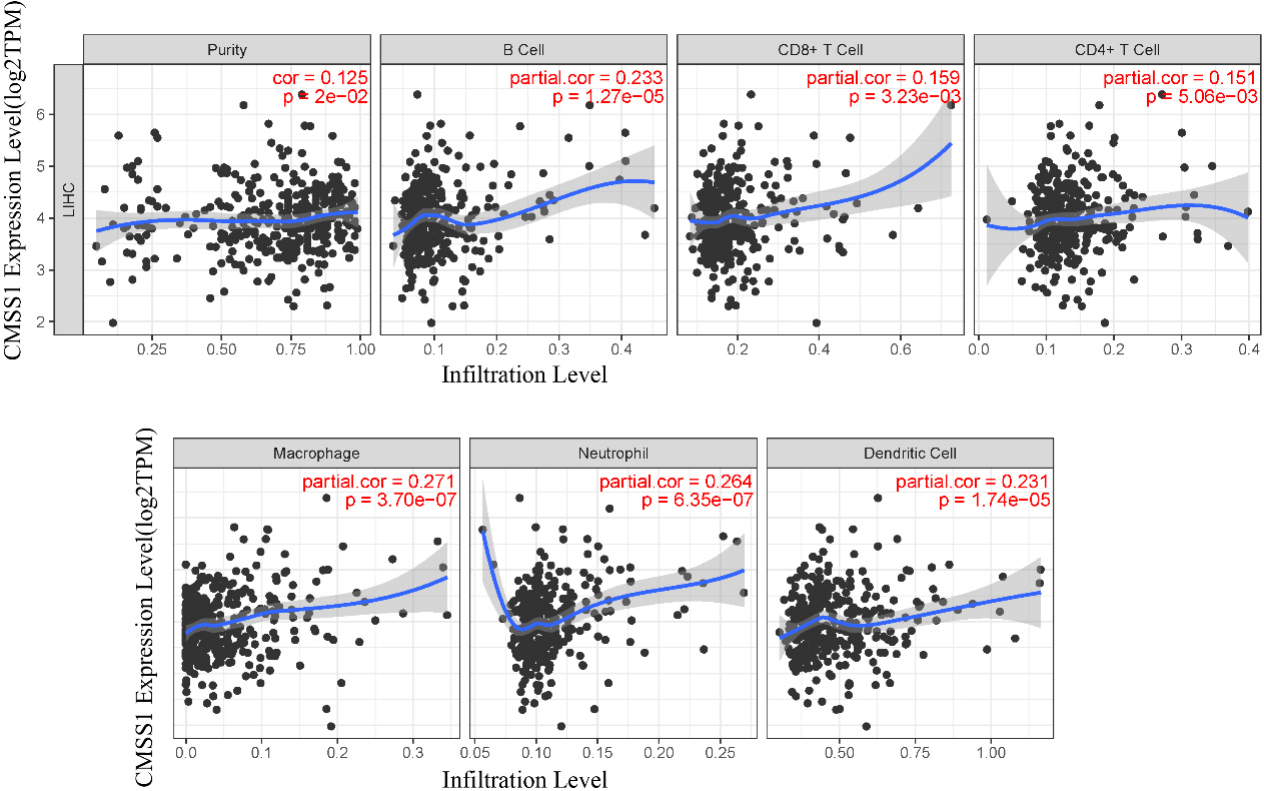
Correlation of CMSS1 with six major types of infiltrating immune cells in LIHC.

### 3.3 MiRNAs and transcription factors associated with CMSS1 expression in LIHC

To further explore the regulatory network of CMSS1 in LIHC, we identified some miRNAs and transcription factors that were statistically associated with CMSS1 expression levels by GSEA analysis. The miRNAs that were most associated with CMSS1 were miR-519, miR-527, miR-323, miR-25, miR-32, miR-92, miR-363 and miR-367.The transcription factors most associated with CMSS1 expression in LIHC were FREAC4, E2F1DP1, E2F1DP2 and E2F4DP2. (Table 1).

### 3.4 Correlation of CMSS1 with infiltrating immune cells in LIHC

We comprehensively analyzed the association of CMSS1 gene expression levels with infiltrating immune cells (B cells, CD8+ T cells, CD4+ T cells, macrophages, neutrophils, and dendritic cells) using the TIMER database (Fig. 5). The results showed that CMSS1 expression was associated with B cells (*Cor = 0.125, P= 2e-02*), CD4+ T cells (*Cor = 0.151, P= 5.06e-03*), CD8+ T cells (*Cor = 0.159, P= 3.23e-03*), macrophages (*Cor = 0.159, P= 3.23e-03*), neutrophils (*Cor = 0.264, P= 6.35e-07*), macrophages (*Cor = 0.271, P= 3.70e-07*) and dendritic cells (*Cor = 0.231, P= 1.74e-05*). Subsequently, we used a multifactorial analysis of the effects of CMSS1 and immune infiltrating cells on the survival of LIHC and found that CMSS1 was an independent influencing factor on the OS of LIHC patients (Table 2).

### 3.5 Protein atlas

**Figure 6.**
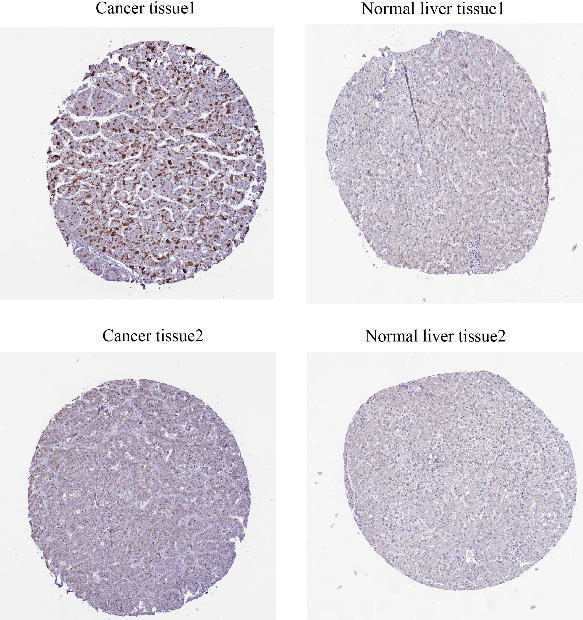
Immunohistochemistry suggested that the expression of CMSS1 was higher in LIHC tissues than in normal tissues.

From the figure, we can see that CMSS1 is mainly expressed in the nucleus, showing moderate intensity staining, while there is basically no expression of CMSS1 in normal hepatocytes (Fig. 6).

## 4. Discussion

LIHC is one of the common malignant tumors. Although it has been improved to some extent, the prognosis of LIHC is still not optimistic. Early diagnosis and early treatment are the key to further improve the prognosis of these patients [16, 17]. At present, identifying more biomarkers is one of the important strategies to achieve early diagnosis and early treatment. RNA-binding protein (RBP) is a key regulator of post-transcriptional gene expression, and it is a protein that can bind to RNA [18]. Many RBPs have been reported to play an important role in promoting cancer development [19, 20, 21, 22, 23]. CMSS1 is an RBP, and whether CMSS1 has a regulatory role in liver cancer is unknown. To clarify the diagnostic and prognostic role of CMSS1 in LIHC, we performed a bioinformatic analysis of the impact of CMSS1 on the clinicopathological features and prognosis of LIHC in public sequencing databases, and explored the relevance of CMSS1 in LIHC.

We first explored the differences in CMSS1 expression in LIHC cancer tissues and normal tissues. The results suggested that the expression level of CMSS1 mRNA in LIHC was significantly higher in cancer tissues than in normal tissues. This suggested that CMSS1 may be a biomarker for the diagnosis of LIHC. In addition, CMSS1 has been found to correlate with clinical or pathological features of LIHC. First, CMSS1 was associated with clinical stage, with higher levels of CMSS1 expression at more advanced clinical stages. Interestingly, we also found that the expression of CMSS1 was significantly higher in TP53-mutated LIHC than in non-TP53-mutated LIHC. TP53, a tumor suppressor gene, has been reported to be one of the most frequently mutated genes in hepatocellular carcinoma [24]. TP53 mutants are reported to be involved in regulating cell cycle arrest, apoptosis, stem cell-like traits, senescence and DNA repair in LIHC [25, 26]. In addition, patients with LIHC with TP53 mutations have a shorter overall survival time compared to patients with TP53 wild type [26]. Therefore, it is likely that CMSS1 exerts its role in regulating LIHC through the TP53 signaling pathway, but the molecular mechanisms involved need to be further elucidated. Finally, we found that the expression level of CMSS1 was significantly associated with both relapse-free survival and overall survival of patients. This suggests that CMSS1 can be used not only as a molecular marker for the diagnosis of LIHC, but also as a molecular marker for predicting the prognosis of LIHC.

Gene enrichment analysis showed that CMSS1 was mainly involved in ribonucleoprotein complex biogenesis, rRNA metabolism, ncRNA processing, translation initiation, RNA localization, and RNA catabolism. This is consistent with the role of CMSS1 as an RNA-binding protein. Then we performed enrichment analysis of CMSS1 using GSEA and obtained several miRNAs and transcription factors significantly associated with it. We found that CMSS1 in LIHC is associated with transcription factors including E2F1 and E2F4. The E2F family plays a critical role in controlling cell cycle and tumor suppressor proteins that mediate cell proliferation and p53-dependent/independent apoptosis [27]. This also suggests that CMSS1 may be associated with the value-added or apoptosis of LIHC.

It has been reported that miRNAs are associated with tumor proliferation, apoptosis, migration, invasive recurrence, and metastasis [28, 29]. These miRNAs can affect the post-transcriptional regulation of gene expression, which in turn affects tumor progression. We therefore analyzed the miRNAs associated with CMSS1 expression. as a result, we identified several miRNAs associated with CMSS1 expression, such as miR-519, miR-527, and miR-25. Indeed, MIR-519, MIR-527 and miR-25 could be used as diagnostic and prognostic markers of malignancy.

Our study inevitably has some limitations. First, our results were highly dependent on bioinformatic analysis, but the database provided less information on patient treatment, so we were unable to explore whether CMSS1 expression levels had an impact on treatment response in patients with LIHC. Second, although our results suggest that CMSS1 may be a potential diagnostic and prognostic marker for hepatocellular carcinoma. We did not perform in vivo and in vitro studies to verify whether CMSS1 is a true oncogenic gene. The results of this study still need to be validated by extensive experiments. Third, the present study did not perform in vivo and in vitro studies to clarify the specific mechanism of CMSS1 in LIHC, and more studies are needed to further explore the mechanism of CMSS1. Fourth, this study only found upregulated CMSS1 in hepatocellular carcinoma tissues, but did not examine whether altered levels of the gene were also present in the blood. We attempted to explore the sequencing data of blood mRNAs from hepatocellular carcinoma patients in the GEO database, but unfortunately did not find data that could be used to analyze blood mRNA-related data. It is expected that future studies will further explore the expression level of CMSS1 in liver cancer blood. Fifth, more research is indeed needed to further clarify whether CMSS1 can be a biomarker. The present study is only a preliminary indication that CMSS1 could be a potential molecular marker.

In conclusion, the present study suggests that CMSS1 is a potential molecular marker for the diagnosis and prognostic determination of LIHC. Meanwhile, our results suggest that CMSS1 is associated with the composition of the immune microenvironment of LIHC. However, the present study was based on the results of bioinformatic analysis, and more in vivo and in vitro experimental studies are needed to validate it subsequently, which will further elucidate the regulatory mechanism of CMSS1 in LIHC.

## Data availability

All data generated or analyzed in this study are included in this article.

## Competing interests

The authors declared that they have no competing interests.

## Authors’ contributions

Cheng Chen, Jincai Wu, and Caiming Wang are responsible for the research design. Cheng Chen and Wei Liu are responsible for collecting, analyzing, and interpreting the data. Cheng Chen, Jiacheng Chen, Liang Chen and Xiangxiang Luo are the main contributors to the writing of manuscripts. The final draft was read and approved by all authors.

## Patient consent for publication

Not applicable.
